# Viability of Commercially Available Rapid Test Strips for Mycotoxin Analysis Compared to Chromatographic Methods

**DOI:** 10.3390/toxins18070283

**Published:** 2026-06-29

**Authors:** Klaudia Bucoń, Paweł Skrzydlewski, Robert Kosicki, Magdalena Twarużek

**Affiliations:** Department of Physiology and Toxicology, Faculty of Biological Sciences, Kazimierz Wielki University, Chodkiewicza 30, 85-064 Bydgoszcz, Poland; klabuc@ukw.edu.pl (K.B.); paskrzy@ukw.edu.pl (P.S.); robkos@ukw.edu.pl (R.K.)

**Keywords:** mycotoxins, contaminants, chromatographic methods, rapid tests

## Abstract

Mycotoxins are toxic secondary metabolites produced primarily by molds of the genera *Aspergillus*, *Fusarium*, and *Penicillium*. These widespread food and feed contaminants can cause significant risks to human and animal health. The aim of this study was to compare the performance of reference chromatographic methods (high-performance liquid chromatography coupled with either fluorescence detection (HPLC-FLD) or tandem mass spectrometry (HPLC-MS/MS)) with two commercially available rapid tests from two manufacturers. To that end, 90 randomly selected grain samples (barley n = 10, wheat n = 21, triticale n = 10, maize n = 49) collected in 2025 were analyzed for deoxynivalenol (DON), zearalenone (ZEN), ochratoxin A (OTA), and the sum of T-2 and HT-2 toxins. None of the samples exceeded the maximum levels established by the European Union (EU); however, widespread contamination with one or more mycotoxins was observed. Results showed that although rapid test strips offer advantages such as low cost, short analysis time, and operational simplicity, their considerably higher limits of detection and quantification values make them unsuitable for advanced laboratory analysis. Therefore, HPLC-FLD and HPLC-MS/MS remain the gold-standard methods for reliable, sensitive, and precise mycotoxin determination.

## 1. Introduction

One of the major challenges of modern agriculture is the presence of toxic substances of both anthropogenic and natural origin. Among naturally occurring contaminants, cotoxins are of particular importance due to their widespread occurrence and adverse effects on human and animal health [1]. These secondary metabolites, produced mainly by molds of the genera *Penicillium*, *Aspergillus*, and *Fusarium*, may develop during plant growth and grain storage. Recent studies indicate that approximately 25% of global agricultural production contains mycotoxins at levels exceeding European Union (EU) regulatory limits, while 60–80% of commodities contain detectable residues [1].

The occurrence of mycotoxins is strongly influenced by climatic and environmental conditions. Climate change alters the distribution of mycotoxin-producing molds, increasing the prevalence of species such as *Fusarium graminearum* and *Aspergillus flavus* in Europe, thereby raising the risk of fumonisin and aflatoxin contamination [2,3]. Among more than 400 known mycotoxins [4], trichothecenes, zearalenone (ZEN), and ochratoxin A (OTA) are most relevant in Europe due to their teratogenic, nephrotoxic, and endocrine-disrupting effects [4]. Their occurrence depends on seasonal weather variability, storage conditions, and agricultural practices [5,6].

Food and feed safety regulations define maximum levels for selected mycotoxins; however, they cover only a fraction of known compounds and do not fully address potential combined toxic effects. Consequently, there is a continuous need for reliable and sensitive analytical strategies for routine monitoring of food and feed. High-performance liquid chromatography coupled with fluorescence (HPLC-FLD) or tandem mass spectrometric detection (HPLC-MS/MS) is widely regarded as a reference approach because of its sensitivity, selectivity, and robustness. However, these methods are limited by high cost, long analysis time, and the requirement for specialized instrumentation and trained personnel. In contrast, lateral flow immunoassays (LFIA) have gained increasing attention as rapid screening tools due to their simplicity, low cost, and suitability for on-site analysis, although their analytical reliability compared with reference methods remains under evaluation.

Against this background, the aim of the present study was to evaluate and compare the performance of two reference chromatographic methods (HPLC-FLD and HPLC-MS/MS) with two commercially available LFIA rapid test systems for the determination of selected mycotoxins (deoxynivalenol (DON), ZEN, OTA, and T-2/HT-2 toxins) in cereal grain samples. The comparison is particularly relevant in view of the increasing implementation of rapid screening tools in food and feed monitoring systems. Ninety grain samples collected in 2025 were analyzed. Although none of the samples exceeded EU limits, widespread low-level contamination was observed. The results are discussed in terms of method agreement, analytical performance, and the suitability of rapid immunoassays as screening tools compared with chromatographic reference techniques.

### 1.1. Mycotoxins

OTA is produced mainly by *Aspergillus ochraceus*, *A. carbonarius*, and *Penicillium verrucosum*. Its production is favored by moderate temperatures and high humidity, which are observed in stored grains, grapes, and feed [7]. Due to its high chemical stability, OTA is not effectively removed by standard food processing methods [8]. In humans, OTA is strongly nephrotoxic, induces oxidative stress, disrupts mitochondrial function, and exhibits genotoxic and carcinogenic potential [9]. Studies in mice show that even short-term exposure leads to apoptosis in liver and kidney cells [10]. In livestock, OTA contributes to reduced productivity and weakened immunity [11]. In an environmental context, climate change, especially temperature increases and changes in humidity, may increase the risk of exposure to OTA [12].

ZEN is produced mainly by *Fusarium graminearum*, *F. culmorum*, *F. cerealis*, and *F. nivale* [13]. Its production is favored by moderate temperatures and high humidity common in cereal crops [14]. ZEN acts as a potent xenoestrogen, binding to estrogen receptors and causing reproductive disorders in animals. Even low doses induce changes in receptor expression, vulvar edema, and apoptosis in reproductive tissues [15]. At the cellular level, ZEN affects the development of egg cells and granulosa cells, disrupts steroidogenesis and ovarian follicle growth processes, which affects fertility [16]. Ecologically, ZEN may serve as a competitive defense metabolite for *Fusarium*, protecting it from other molds or fungal parasites [17].

T-2 and HT-2 toxins are type A trichothecenes produced by *Fusarium sporotrichioides*, *F. poae*, and *F. langsethiae* [18]. Their production is promoted by humid conditions and moderate temperatures [19]. These toxins are extremely potent inhibitors of protein synthesis and exhibit immunosuppressive and hematotoxic effects. In animals, they caused reduced feed intake, weight loss, vomiting, diarrhea, gastrointestinal bleeding, as well as skin damage (necrosis) [20]. At the cellular level, T-2 induces oxidative stress, deoxyribonucleic acid (DNA) damage, apoptosis, and protein translation disorders, which cause cell death [21]. T-2 can penetrate the skin, mucous membranes of the gastrointestinal tract, and respiratory tract, which increases the risk of exposure in humans [22]. In an environmental context, the presence of T-2 and HT-2 in cereals poses a serious threat, as they are common contaminants of cereals, especially oats, barley, and wheat [23].

DON, a type B trichothecene, is produced mainly by *Fusarium graminearum* and *F. culmorum* [24]. Its production is favored by cool or moderate temperatures combined with high humidity [25]. In humans, DON causes gastrointestinal disorders such as nausea, vomiting, and diarrhea, and stimulates inflammation and oxidative stress in intestinal cells [26]. In animals, it suppresses immune function and contributes to reduced productivity [27]. Due to its high thermal and chemical stability, DON persists during food processing, leading to chronic dietary exposure [24].

### 1.2. Analytical Methods

High-performance liquid chromatography (HPLC) is one of the most widely used analytical techniques for separating complex mixtures [28]. The separation mechanism is based on differences in the interaction of analyte molecules with the stationary phase and their solubility in the mobile phase, which, flowing through the column under high pressure, causes the migration of compounds with different retention times [29]. The migration of individual molecules is only possible when they are in the mobile phase, which results in characteristic detection signals. The separation of components takes place inside the analytical column, and the recorded signals are interpreted by a detector, most often ultraviolet-visible spectrophotometric (UV-Vis), photodiode array (PDA), or diode array detector DAD) or fluorescence detector (FLD) [30,31,32]. HPLC enables the analysis of very low concentrations of compounds present in complex samples, which significantly increases the research capabilities of an analytical laboratory. The most commonly used technique is reversed-phase chromatography (RP-HPLC), which is the opposite of normal-phase chromatography. The use of a nonpolar stationary phase and a polar mobile phase enables the effective separation of a wide range of both polar and nonpolar compounds, making this method widely used in environmental, pharmaceutical, biological, and food analysis [33]. In the HPLC method, the separation of analytes takes place in a column filled with a suitably selected stationary phase, most often with reversed polarity (RP), which enables effective separation of both polar and nonpolar compounds [34]. (HPLC-FLD) exhibits high sensitivity and selectivity for naturally fluorescent compounds, such as aflatoxins (AF) and OTA, as well as compounds requiring prior derivatization, including fumonisins [35]. HPLC-FLD detection, thanks to its low noise level and high resistance to matrix interference, remains particularly valued in routine analyses, where repeatability and relatively low cost of determinations are key [36]. Despite its numerous advantages, HPLC-FLD is a selective method only for selected groups of toxins and requires additional sample preparation steps, which makes it less versatile for the simultaneous determination of multiple classes of mycotoxins [37]. For this reason, HPLC-MS/MS is increasingly used and is currently regarded as the reference method for the simultaneous determination of multiple mycotoxins. This technique allows for the simultaneous, precise, and sensitive determination of several dozen toxins in a single run, without the need to perform derivatization reactions on most compounds [38]. The use of selective multiple reaction monitoring (MRM) and isotope-labeled standards effectively minimizes matrix effects, thereby significantly improving analytical accuracy even in complex matrices such as cereals, maize, and processed plant products [39]. As a result, HPLC-MS/MS remains an irreplaceable technique in legislative analyses, food chain monitoring studies, and in assessing consumer exposure to multiple toxins present in a single sample.

However, the HPLC technique, especially coupled with MS/MS, also has several downsides. First of all, it requires expensive and relatively large equipment. It also required specialized personnel to operate, since those techniques are very advanced and sophisticated. Therefore, alternative solutions, which allow for rapid analysis on site, without specialized training, have gained much popularity in recent years.

One such solution is rapid strip tests, which allow for quick mycotoxin analysis directly on a farm or even in a field. These tests are based on immunochromatography, a type of immunoassay employing antibodies for the detection of target analytes or their metabolites. This approach enables efficient screening of a large number of samples in a short time frame [40]. LFIA is commonly used in such applications. The principle of LFIA is based on the capillary-driven flow of a liquid sample or extract through a multi-layered test strip. A typical test strip consists of a sample pad, an absorbent pad, nitrocellulose, and a test membrane (with two distinct sections: T-test and C-control). LFIA can be divided into competitive immunochromatography and double-antibody sandwich immunochromatography. Those two methods are different due to the relationship between the quantity of analyte in the sample and the intensity of the signal on the test strip. In the competitive approach, the more analyte is in the sample, the lighter the color of the test (T) section on the test membrane. In double antibody sandwich immunochromatography, the more analyte is in the sample, the more intense the color of the test (T) section on the test membrane [41]. The commercial lateral flow assays used in this study were manufacturer-validated for specific mycotoxins in cereal matrices, according to the intended use defined by the manufacturer. These tests are designed for qualitative or semiquantitative screening purposes and are not intended for confirmatory analysis. The validation performed by the manufacturer included evaluation in relevant food matrices such as cereals and feed, and the reported limits of detection are typically in the low µg/kg range for solid samples (corresponding to ng/mL levels in extract solutions). Therefore, LFIA provides rapid on-site screening capability, while chromatographic methods such as HPLC-FLD or HPLC-MS/MS remain necessary for confirmatory quantification due to their significantly lower limits of detection (LOD) and limits of quantification (LOQ) values.

## 2. Results

For most samples, mycotoxin concentrations were detected above LOQ values for HPLC-FLD or HPLC-MS/MS methods. They were, however, below the LOD or LOQ value for LFIA strips analysis. In Table 1 are presented results for analysis performed with all three methods where results were different than zero. Statistical analysis for each mycotoxin is presented in Table 2, Table 3, Table 4 and Table 5.

## 3. Discussion

Agricultural production in Poland is among the largest in the EU. It was ranked third in cereal production, being ahead of Romania (20.8 million tons) with 35.2 million tons. France took first place in this ranking (64.2 million tons), followed by Germany (42.5 million tons) [42]. Data shows a recent shift in terms of the usage of harvested grains. Use of grains for feed production increased, and use of unprocessed grains for animal feeding decreased, mainly due to a reduction in swine production and growth in poultry farming [43].

In the present study, DON was detected in 77 samples using HPLC-MS/MS, while LFIA tests showed markedly lower detection rates (13 positives for reader no. 1 and 9 for reader no. 2). The highest DON concentration of 1670 µg/kg reached in maize using reader no. 1. According to EU recommendation, DON concentration should not exceed 8000 μg/kg for cereal feed materials and 12,000 μg/kg for maize feed materials [44]. None of the examined samples exceeded the EU-recommended limits. In 2021, Babič et al. examined 433 cereal samples from Slovenia and found DON in 32% of them, with the highest concentration of 4082 µg/kg [45]. Bertuzzi et al. analyzed 80 samples of durum wheat, 80 samples of common wheat, and 81 maize samples. DON was present in 96.2% of durum wheat samples, 57.5% of common wheat samples, and 80.2% of maize samples, rarely exceeding 500 µg/kg [46]. Recent evidence indicates that mycotoxin occurrence should be interpreted within a broader food matrix and ecological context. Buonsenso et al. (2026) demonstrated the detection of multiple Penicillium-derived toxins in nuts marketed in Italy using HPLC-MS/MS, confirming that the co-occurrence of fungal secondary metabolites extends beyond cereals and also affects other plant-derived food matrices [47]. In addition, Garello et al. (2024) showed that *Penicillium* spp. genomes contain multiple secondary metabolite biosynthetic gene clusters, suggesting a high inherent potential for simultaneous mycotoxin production in different food ecosystems [48]. These findings support the concept of multi-mycotoxin exposure as a general phenomenon in food chains and reinforce the relevance of multianalyte analytical strategies.

Regarding LFIA tests, in 2010, Xu et al. developed a strip test using monoclonal antibodies labeled with colloidal gold. Their limit of detection for DON in wheat and maize samples was set at 50 µg/kg, which is considerably lower than the recommended limit set by both the EU government and the Chinese government [49]. In another study, Huang et. al. developed an immunochromatographic strip test for simultaneous detection of DON and fumonisin B1. This method exhibited a LOD of 20 ng/mL, which is substantially lower than the maximum levels recommended by the EU [50]. In their study, Hou et al. developed a test strip for simultaneous analysis of ZEN, DON, and fumonisin B1. LOD for DON was established at 12.5 ng/mL, and the results obtained from this test were in agreement with the results obtained from the HPLC-MS/MS method [51]. A marked discrepancy between chromatographic and LFIA-based results was observed in the present study, likely reflecting differences in analytical sensitivity, matrix effects, and extraction efficiency. HPLC-MS/MS provides substantially lower limits of detection and quantification, enabling detection of trace-level contamination that may not be captured by immunochemical assays. In contrast, LFIA performance depends on antibody affinity, matrix complexity, and reader variability, which may lead to underestimation of positive samples, particularly near cut-off values [43,44]. Differences in analytical performance are further reflected in LOD/LOQ disparities between methods. While chromatographic techniques achieve detection at µg/kg–ng/kg levels, LFIA assays are typically reported in ng/mL for extract solutions, which translates into reduced matrix sensitivity in real samples. Although improved immunoassay formats (e.g., fluorescence-enhanced or nanoparticle-based systems) can partially bridge this gap, their robustness in complex cereal matrices remains limited [41].

ZEN was detected in 82 samples using HPLC-MS/MS, 14 samples using reader no. 1, and 17 samples using reader no. 2, with maximum concentration in maize exceeding 500 μg/kg using reader no. 2. EU recommended maximum concentration level of 3000 μg/kg in maize materials and 2000 μg/kg in cereal materials [44]. Of all samples analyzed, none exceeded recommended EU levels. In their study, Babič et al. analyzed 433 ground cereal samples. ZEN was present in 5% of them, with the highest concentration of 149 µg/kg [45]. Drakopoulos et al. analyzed barley samples from Switzerland. Out of 253 samples, ZEN was found in 38% of them, with a maximum concentration of 341 μg/kg [52]. In another study, Zachariasova et al. investigated ZEN concentration in 8 maize samples, 21 wheat samples, and 26 barley samples. Results showed the maximum concentration level for maize to be at 159 μg/kg, for wheat to be at 131 μg/kg, and for barley to be at 17 μg/kg [53]. Regarding LFIA tests, Wang et al. developed a time-resolved fluorescence immunochromatographic (TRFI) test strip designed for ZEN detection in cereals. They tested this method in both laboratory and real-life conditions, reaching a LOQ of 20 µg/kg [38]. In their study, Hou et al. developed a test strip for simultaneous analysis of ZEN, DON, and fumonisin B1. LOD for ZEN was established at 6 ng/mL, and the results obtained from this test were in agreement with the results obtained from the HPLC-MS/MS method [51]. In another study, a rapid test strip was developed for simultaneous detection of ZEN and fumonisin B1 in maize and wheat samples. LOD for their method was set at 6 ng/mL, and the obtained results were in agreement with the results from the HPLC-MS/MS method [54].

In this study, OTA was detected in 9 samples using HPLC-FLD, 41 samples using reader no. 1, and 5 samples using reader no. 2, with the highest concentration of 94 µg/kg in wheat using HPLC-FLD. EU recommended maximum concentration level of 250 μg/kg for cereal feed materials (including maize) [44]. None of the samples tested exceeded the EU-recommended maximum concentration levels. In their study, Zachariasova et al. analyzed 21 wheat samples. Those samples originated from the United Kingdom (UK) and the Czech Republic, with the maximum concentrations of 58 µg/kg [53]. In their study, Bryła et al. analyzed 99 wheat samples from Poland. They found that two samples were contaminated with OTA, with a maximum concentration of 7 μg/kg [55]. In another study, conducted by Ibáñez-Vea et al., 123 barley samples from Spain were examined. OTA was found in 71 samples, with a maximum concentration of 3.53 μg/kg [56]. In their study, Kos et al. analyzed OTA in 204 maize samples originating from Serbia. They found that 23 samples were contaminated with OTA with a maximum concentration of 318 μg/kg [57]. Regarding LFIA tests, Zheng et al. developed a dual-signal LFIA using both colorimetric and fluorescent detection. This assay exhibited LOD values of 0.084 ng/mL and 0.032 ng/mL for colorimetric and fluorescent analysis, respectively [58]. In another study, an immunostrip assay was developed for simultaneous detection of AF B1 and OTA. This test provided a visual detection limit of 5 ng/mL for OTA [59]. In their study, they developed lateral flow strips using mimotope peptides. They obtained an LOD value of 10 ng/mL for wheat and maize samples [60].

The sum of T-2 toxin and HT-2 toxins was detected in 51 samples using HPLC-MS/MS, 23 using reader no. 1, and only 4 using reader no. 2. The highest concentration measured chromatographically was 146.8 μg/kg in maize, well below the EU recommended limit of 500 µg/kg for cereal feed materials (including maize) [61]. None of the examined samples exceeded the EU-recommended limits. In their study, Haidukowski et al. analyzed 141 durum wheat samples. They found T-2 and HT-2 toxins in 76 samples with a maximum concentration of 486 μg/kg [62]. In another study, Janić Hajnal et al. examined 204 maize samples from Serbia. T-2 was detected in 96 samples and HT-2 toxin in 40 samples, with maximum concentrations reaching 99 μg/kg and 178 μg/kg, respectively [63]. In their study, Macri et al. examined 121 cereal samples from Romania for HT-2 toxin. 21 samples were contaminated with HT-2 toxin, with maximum concentrations of 18 μg/kg [64]. Regarding LFIA- based methods, Chen et al. developed two immunochromatographic test strips based on signal amplification and selenium nanoparticles. They reached a sensitivity of 1 ng/mL and 0.25 ng/mL for the signal amplification test and the selenium nanoparticles test, respectively [65]. In another study, a gold nanoparticle-based multiplex immuno-strip was developed for simultaneous detection of several mycotoxins in cereals, including T-2 toxin, with an LOD of 0.1 ng/mL [66].

## 4. Conclusions

Food and feed safety is one of the most important issues worldwide. Therefore, numerous governments and organizations, including the EU, place strong emphasis on this issue through regulations establishing maximum permitted concentrations of contaminants in food and feed commodities. Highest agricultural standards are essential during crop cultivation, harvesting, and storage, particularly for cereals, maize, and other food commodities. Mycotoxins are among the most serious threats to food and feed safety. Due to the climate, ZEN, DON, OTA, T-2, and HT-2 toxins are the most commonly occurring mycotoxins in Poland, usually occurring simultaneously. However, due to climate change, we can observe a shift in this trend in favor of other mycotoxins (such as AF). Since none of the examined samples exceeded EU-recommended limits, this can indicate that, although widespread, mycotoxin contamination remains within manageable levels. On the other hand, the EU regulates only a few of over 400 mycotoxins characterized to date. Those limits also do not take into account the potentially synergistic effects of co-occurring mycotoxins. Although HPLC-FLD or HPLC-MS/MS methods remain the gold standard for mycotoxin analysis, due to their superior sensitivity and precision, they require specialized personnel to perform and are very costly. In response to that, rapid tests are becoming more and more popular among farmers and food and feed producers. In the last decade, the number of commercially available tests has increased considerably, proving their growing popularity. Also, their sensitivity and precision are constantly improving. This study showed that rapid strip tests are suitable for on-site screening analysis in terms of potential health risks, making mycotoxin screening a more common practice, which in turn can contribute to the overall improvement of food and feed safety. These tests, however, are not suitable for specialized laboratory analysis or experiments, due to considerable higher LOD and LOQ values, compare to HPLC-MS/MS methods. It is also worth mentioning that suspicious results obtained from rapid strip tests should always be confirmed by more specialized analysis. Taking into account serious health risks, as well as economic losses associated with mycotoxin contamination, continuous monitoring of raw materials, as well as food and feed, is crucial. A combination of strict regulatory oversight, regular testing, and preventive agricultural and storage practices can help maintain food and feed safety across Europe.

## 5. Materials and Methods

### 5.1. Samples and Sampling Procedure

A total of 90 samples (barley n = 10, wheat n = 21, triticale n = 10, maize n = 49) were randomly selected from samples delivered to the Laboratory of Mycotoxin Analysis in 2025. Samples were provided by farmers, feed producers, and veterinary doctors from all over Poland. The initial weight of the samples was approximately 1 kg each. Each sample was analyzed using both chromatographic methods (HPLC-FLD or HPLC-MS/MS) and two separate LFIA strip tests. The presence of DON, ZEN, OTA, and the sum of T-2 and HT-2 toxins was determined, depending on the availability of validated matrices for each analysis type.

### 5.2. Chemicals

All solvents and reagents were of analytical or chromatographic grade. Acetonitrile (ACN), methanol (MeOH), sodium chloride, acetic acid (CH_3_COOH), ammonium acetate (CH_3_COONH_4_), and potassium bromide were obtained from Merck (Darmstadt, Germany). Certified reference standards, including 13C-labeled internal standards for ZEN, DON, T-2 toxin, HT-2 toxin, and OTA, were acquired from Romer Labs Diagnostics GmbH (Tulln, Austria). Deionized water was produced using a Simplicity UV water purification system (Merck).

### 5.3. HPLC-FLD/HPLC-MS/MS Analysis

LOD and LOQ for each mycotoxin were experimentally determined from the signal-to-noise ratio (S/N) obtained for low-concentration fortified samples and were defined as the concentrations corresponding to S/N ratios of 3:1 and 10:1, respectively. Recovery and precision (expressed as repeatability (% RSD)) were assessed by analyzing samples spiked at different concentration levels in triplicate (Results presented in Table 6).

#### 5.3.1. OTA—Sample Preparation

OTA was extracted as described by Błajet-Kosicka et al. In short, 12.5 g of sample was homogenized for 2 min with 50 mL ACN:H_2_O (60:40, *v*/*v*). The sample was then filtered. Next, 5 mL of extract was diluted with 55 mL of phosphate-buffered saline (PBS) and filtered again. 48 mL of diluted and filtered extract was applied to an Ochraprep^®^ immunoaffinity column (Rhone Diagnostic Technologies Ltd., Glasgow, UK). Next, the column was washed with 20 mL of water and dried with air. OTA was eluted using 1.5 mL MeOH: CH_3_COOH (98:2, *v*/*v*) and 1.5 mL of water [46].

#### 5.3.2. OTA—Chromatographic Analysis

OTA was analyzed using HPLC-FLD. Chromatographic analyses were performed using a LaChrom ELITE system (Merck-Hitachi, Darmstadt, Germany), equipped with a LiChrospher 100 RP-18 analytical column (250 × 4.0 mm, 5 μm). The mobile phase consisted of ACN:2%CH_3_COOH (70:30, *v*/*v*), delivered at a flow rate of 1.0 mL/min, with an injection volume of 50 μL. LOD and LOQ for OTA were 0.13 and 0.4 μg/kg, respectively.

#### 5.3.3. Fusarium Mycotoxins—Sample Preparation for ZEN and Trichothecenes

Trichothecenes and ZEN were extracted by homogenizing 12.5 g of sample with 50 mL ACN:H_2_O (80:20, *v*/*v*). After homogenization, the extract was filtered. Next, 4 mL of extract was taken for further preparation, and 40 μL of internal standard solution (13C-ZEN, c = 1000 μg/L) was added and mixed thoroughly. Then, the extract was applied to a Bond Elut^®^ Mycotoxin column (Agilent, Santa Clara, CA, USA). Then, 2 mL of extract was taken for further preparation, and 50 μL of internal standard solution (13C-T2, c = 250 μg/L; 13C-HT-2, c = 250 μg/L; 13C-DON, c = 2500 μg/L) was added to the extract. The mixture was then dried using nitrogen at 50 °C. Finally, reconstitution was performed with 495 μL of MeOH:H_2_O (20:80, *v*/*v*).

#### 5.3.4. Fusarium Mycotoxins—Chromatographic Analysis of ZEN and Trichothecenes

ZEN and trichothecenes were analyzed using HPLC-MS/MS detection. The chromatographic separation was performed using Shimadzu Nexera with Gemini-NX-C18 chromatographic column (150 × 4.6 mm, 3 μm; Phenomenex, Torrance, CA, USA) using 1% CH_3_COOH in H_2_O (mobile phase A) and MeOH (mobile phase B) with addition of 5 mM CH_3_COONH_4_ to both phases at a flow rate of 0.5 mL/min with injection volume of 7 μL. Mass spectrometry detection was performed using API 4000 (Sciex, Marlborough, MA, USA). LOD and LOQ for ZEN were 0.07 and 0.2 μg/kg, for T-2 toxin were 0.2 and 0.6 μg/kg, for HT-2 toxin were 0.7 and 2 μg/kg, and for DON were 1 and 3 μg/kg, respectively.

### 5.4. LFIA Analysis of Mycotoxins

LFIA analysis of mycotoxins was performed using two different strip tests and strip readers commercially available from two different manufacturers, both being global leaders in the field of food and feed safety. LOD and LOQ values are presented in Table 7. Summary of examined mycotoxins and validated matrices are presented in Table 8.

#### 5.4.1. Mycotoxin Analysis—Reader No. 1

All procedures were performed according to the manufacturer’s instructions. In short, 10 g of well-ground samples was homogenized with the addition of 50 mL of distilled water and 1 bag of buffer provided by the manufacturer, as part of the analysis kit. Then, the sample was shaken vigorously for 2 min. Next, the filtered sample was added to the dilution buffer (the exact amount of sample and dilution buffer depended on the matrix type and the mycotoxin analyzed). Then, the extract was centrifuged for 30 s (2000× *g*). Then, 100 µL of extract was transferred onto the sample pad of a strip previously inserted in the reader (to provide the right temperature for the analysis).

#### 5.4.2. Mycotoxin Analysis—Reader No. 2

All procedures were performed according to the manufacturer’s instructions. In short, 20 g of well-ground samples was homogenized with the addition of distilled water in an appropriate ratio (the exact ratio depended on matrix type and mycotoxin analyzed). Then the sample was shaken vigorously. Next, the extract was centrifuged for 30 s (2000× *g*). Then, 100 µL of buffer was added to 100 µL of extract. After 2 min, a strip was inserted into the extract. After a few minutes (exact time depended on matrix type and mycotoxin analyzed), a strip was transferred into the reader.

## Figures and Tables

**Table 1:** Results for analysis performed by all three methods with results different than 0. Analysis with quantifiable results from all three methods is emboldened.

Mycotoxin	Matrix	HPLC-FLD/HPLC-MS/MS [µg/kg]	Reader 1 [µg/kg]	Reader 2 [µg/kg]	SD [µg/kg]
DON	**barley**	**1206**	**1260**	**980**	**146**
DON	wheat	206	<LOD	230	126
DON	**maize**	**944**	**520**	**6** **80**	**214**
DON	**maize**	**845**	**460**	**710**	**195**
DON	**maize**	**696**	**520**	**580**	**89**
ZEN	maize	3.51	<LOQ	67	31.9
ZEN	maize	5.05	<LOQ	58	26.9
ZEN	maize	29.2	<LOQ	<LOD	20.7
ZEN	maize	128	62	<LOD	64
ZEN	**maize**	**68.5**	**58**	**81**	**11.5**
ZEN	maize	19.6	<LOQ	<LOD	20
ZEN	maize	21.6	<LOD	<LOD	12.4
ZEN	maize	13.4	<LOD	<LOD	7.74
ZEN	maize	88.5	84	<LOD	49.8
ZEN	**maize**	**112**	**129**	**160**	**24.3**
ZEN	maize	17.5	54	<LOD	27.6
ZEN	maize	0.37	<LOQ	<LOD	23
ZEN	maize	50	<LOD	55	30.4
ZEN	maize	8.53	<LOD	<LOD	4.92
ZEN	**maize**	**72.4**	**55**	**53**	**10.7**
ZEN	maize	4.93	<LOD	<LOD	2.85
ZEN	maize	4.08	<LOD	<LOD	2.36
ZEN	maize	5.42	<LOD	<LOD	3.13
ZEN	maize	36.9	<LOD	<LOD	21.3
ZEN	maize	0.78	<LOD	<LOD	0.45
ZEN	**maize**	**119**	**57**	**80**	**31.3**
ZEN	maize	3.91	<LOD	<LOD	2.26
ZEN	maize	6.61	<LOD	160	90.5
ZEN	maize	96.9	126	>500	225
OTA	maize	0.85	<LOD	2.5	1.27
OTA	**maize**	**0.9**	**5.7**	**4.6**	**2.52**
OTA	**maize**	**0.56**	**1.7**	**1.7**	**0.66**
OTA	**maize**	**0.68**	**1.8**	**1.6**	**0.6**
T-2 + HT-2	wheat	15.39	<LOD	<LOD	8.89
T-2 + HT-2	wheat	2.71	<LOQ	<LOD	13.7
T-2 + HT-2	wheat	<2	37	<LOD	20.8
T-2 + HT-2	wheat	5.63	<LOQ	<LOD	13.1
T-2 + HT-2	wheat	<2.6	26	<LOD	14.3
T-2 + HT-2	wheat	9.26	32	<LOD	16.5
T-2 + HT-2	maize	27.93	53	<LOD	26.5
T-2 + HT-2	maize	13.69	30	<LOD	15
T-2 + HT-2	maize	128.2	47	<LOD	64.9
T-2 + HT-2	maize	10.81	<LOD	<LOD	6.24
T-2 + HT-2	maize	6.64	<LOD	<LOD	3.83
T-2 + HT-2	maize	3.26	<LOD	<LOD	1.88
T-2 + HT-2	**maize**	**92.8**	**65**	**61**	**17.3**
T-2 + HT-2	maize	11.52	<LOD	<LOD	6.65
T-2 + HT-2	**maize**	**146.8**	**82**	**100**	**33.5**

Analysis with quantifiable results from all three methods is emboldened.

**Table 2:** DON concentration was measured using the HPLC-MS/MS method, as well as both readers. For the calculation, the results below the LOD value were set as zero, whereas the results between the LOD and the LOQ were set as the LOQ value. A positive sample refers to a sample with a result above the limit of detection. n.a.—not applicable, manufacturer did not provide tests for this mycotoxin in given matrix.

			Mean ± SD (µg/kg)	Mean Positive ± SD (µg/kg)	Median (µg/kg)	1st Quartile (µg/kg)	3rd Quartile (µg/kg)	Maximum (µg/kg)
DON	barley	HPLC-MS/MS	131 ± 378	131 ± 378	9.72	3.57	23.4	1206
		Reader no. 1	126 ± 398	1260 ± 0	0	0	0	1260
		Reader no. 2	98 ± 310	980 ± 0	0	0	0	980
	wheat	HPLC-MS/MS	73.3 ± 153	73.3 ± 153	28.4	22.7	58	717
		Reader no. 1	31.5 ± 141	630 ± 0	0	0	0	630
		Reader no. 2	11.5 ± 51.4	230 ± 0	0	0	0	230
	maize	HPLC-MS/MS	297 ± 330	297 ± 330	134	96.5	336	1473
		Reader no. 1	142 ± 316	510 ± 425	0	0	200	1670
		Reader no. 2	158 ± 256	361 ± 280	0	0	145	710
	tricitale	HPLC-MS/MS	181 ± 355	181 ± 355	36.5	19.5	101	1144
		Reader no. 1	56 ± 177	560 ± 0	0	0	0	560
		Reader no. 2	n.a.	n.a.	n.a.	n.a.	n.a.	n.a.

**Table 3:** ZEN concentration was measured using the HPLC-MS/MS method, as well as both readers. For the calculation, the results below the LOD value were set as zero, whereas the results between the LOD and the LOQ were set as the LOQ value. A positive sample refers to a sample with a result above the limit of detection. n.a.—not applicable, manufacturer did not provide tests for this mycotoxin in given matrix.

			Mean ± SD (µg/kg)	Mean Positive ± SD (µg/kg)	Median (µg/kg)	1st Quartile (µg/kg)	3rd Quartile (µg/kg)	Maximum (µg/kg)
ZEN	barley	HPLC-MS/MS	1.95 ± 5.5	2.79 ± 6.53	0.22	0.05	0.40	17.6
		Reader no. 1	0 ± 0	0 ± 0	0	0	0	0
		Reader no. 2	n.a.	n.a.	n.a.	n.a.	n.a.	n.a.
	wheat	HPLC-MS/MS	5.72 ± 11.8	6.03 ± 12.1	1.53	0.40	3.54	49
		Reader no. 1	2.5 ± 11.2	50 ± 0	0	0	0	50
		Reader no. 2	n.a.	n.a.	n.a.	n.a.	n.a.	n.a.
	maize	HPLC-MS/MS	24.4 ± 36.3	25.5 ± 36.7	6.61	2.51	29.2	128
		Reader no. 1	22.2 ± 34.9	61.8 ± 30.5	0	0	40	129
		Reader no. 2	37.7 ± 82	100 ± 109	0	0	58	500
	tricitale	HPLC-MS/MS	3.43 ± 7.12	3.81 ± 7.44	0.75	0.37	1.31	23.0
		Reader no. 1	0 ± 0	0 ± 0	0	0	0	0
		Reader no. 2	n.a.	n.a.	n.a.	n.a.	n.a.	n.a.

**Table 4:** OTA concentration was measured using the HPLC-MS/MS method, as well as both readers. For the calculation, the results below the LOD value were set as zero, whereas the results between the LOD and the LOQ were set as the LOQ value. A positive sample refers to a sample with a result above the limit of detection. n.a.—not applicable, manufacturer did not provide tests for this mycotoxin in given matrix.

			Mean ± SD (µg/kg)	Mean Positive ± SD (µg/kg)	Median (µg/kg)	1st Quartile (µg/kg)	3rd Quartile (µg/kg)	Maximum (µg/kg)
OTA	barley	HPLC-MS/MS	n.a.	n.a.	n.a.	n.a.	n.a.	n.a.
		Reader no. 1	0.2 ± 0.63	0 ± 0	0	0	0	0
		Reader no. 2	n.a.	n.a.	n.a.	n.a.	n.a.	n.a.
	wheat	HPLC-MS/MS	8.55 ± 28.3	94 ± 0	0	0.0	0.0	94
		Reader no. 1	6.88 ± 18.4	8.49 ± 20.3	3	3	3.8	87
		Reader no. 2	0 ± 0	0 ± 0	0	0	0	0
	maize	HPLC-MS/MS	0.47 ± 1.51	2.07 ± 2.71	0	0	0	7.57
		Reader no. 1	1.63 ± 2.73	3.17 ± 3.12	1.5	2.3	2.3	15
		Reader no. 2	0.41 ± 1.04	2 ± 1.3	0	0	0	4.6
	tricitale	HPLC-MS/MS	0 ± 0	0 ± 0	0	0	0	0
		Reader no. 1	2.27 ± 2.41	4.54 ± 0.48	1.9	0	4.55	5
		Reader no. 2	n.a.	n.a.	n.a.	n.a.	n.a.	n.a.

**Table 5:** T-2/HT-2 toxins concentration measured using the HPLC-MS/MS method, as well as both readers. For the calculation, the results below the LOD value were set as zero, whereas the results between the LOD and the LOQ were set as the LOQ value. A positive sample refers to a sample with a result above the limit of detection. n.a.—not applicable, manufacturer did not provide tests for this mycotoxin in given matrix.

			Mean ± SD (µg/kg)	Mean Positive ± SD (µg/kg)	Median (µg/kg)	1st Quartile (µg/kg)	3rd Quartile (µg/kg)	Maximum (µg/kg)
T-2/HT-2 toxins	wheat	HPLC-MS/MS	4.07 ± 3.51	4.52 ± 3.41	2.83	2.60	4.58	15.4
		Reader no. 1	19.4 ± 14	26.4 ± 4.25	25	0	28.5	37
		Reader no. 2	0 ± 0	0 ± 0	0	0	0	0
	maize	HPLC-MS/MS	33.2 ± 34.7	34.2 ± 34.7	25.1	11.0	42.3	147
		Reader no. 1	28.1 ± 35	53.3 ± 30.8	20	0	48	123
		Reader no. 2	10.1 ± 25.7	68 ± 21.5	0	0	0	100

**Table 6:** Mycotoxins were measured, and respective limits of detection (LOD) and quantification (LOQ) were established for HPLC methods.

Toxin	LOD µg/kg	LOQ µg/kg	Spiking Level µg/kg	Recovery [%] ± RSD [%], n = 3	
				Corn Samples	Cereal Samples
DON	1	3	500	93 ± 4	90 ± 2
T-2	0.2	0.6	100	88 ± 5	88 ± 2
HT-2	0.7	2.0	100	90 ± 4	82 ± 7
ZEN	0.07	0.2	50	113 ± 7	110 ± 6
OTA	0.1	0.4	10	96 ± 2	90 ± 6

**Table 7:** LOD and LOQ values for analyzed mycotoxins in four matrices; n.a—not applicable—manufacturer did not provide tests for this mycotoxin in the given matrix.

		Maize		Wheat		Barley		Triticale	
		LOD [µg/kg]	LOQ [µg/kg]	LOD [µg/kg]	LOQ [µg/kg]	LOD [µg/kg]	LOQ [µg/kg]	LOD [µg/kg]	LOQ [µg/kg]
OTA	HPLC-FLD	0.13	0.4	0.13	0.4	0.13	0.4	0.13	0.4
	Reader 1	1	1.5	2	3	1	1.5	3	3
	Reader 2	1.5	1.5	1.5	1.5	n.a.	n.a.	n.a.	n.a.
DON	HPLC-MS/MS	1	3	1	3	1	3	1	3
	Reader 1	100	200	100	200	200	300	200	200
	Reader 2	100	100	100	100	100	100	n.a.	n.a.
ZEN	HPLC-MS/MS	0.07	0.2	0.07	0.2	0.07	0.2	0.07	0.2
	Reader 1	25	40	35	45	45	65	40	40
	Reader 2	50	50	n.a.	n.a.	n.a.	n.a.	n.a.	n.a.
T-2 HT-2	HPLC-MS/MS	T-2-0.2 HT-2-0.7	T-2-0.6 HT-2-2	T-2-0.2 HT-2-0.7	T-2-0.6 HT-2-2	T-2-0.2 HT-2-0.7	T-2-0.6 HT-2-2	T-2-0.2 HT-2-0.7	T-2-0.6 HT-2-2
	Reader 1	15	20	20	25	n.a.	n.a.	n.a.	n.a.
	Reader 2	50	50	50	50	n.a.	n.a.	n.a.	n.a.

n.a.—not applicable.

**Table 8:** Summary of validated matrices for each of the analyzed mycotoxins.

		OTA	DON	ZEN	T-2 + HT-2
maize	HPLC	yes	yes	yes	yes
	Reader 1	yes	yes	yes	yes
	Reader 2	yes	yes	yes	yes
wheat	HPLC	yes	yes	yes	yes
	Reader 1	yes	yes	yes	yes
	Reader 2	yes	yes	no	yes
barley	HPLC	yes	yes	yes	yes
	Reader 1	yes	yes	yes	no
	Reader 2	no	yes	no	no
triticale	HPLC	yes	yes	yes	yes
	Reader 1	yes	yes	yes	no
	Reader 2	no	no	no	no

## Data Availability

The original contributions presented in this study are included in the article. Further inquiries can be directed to the corresponding authors.
